# Reduced white matter integrity and disrupted brain network in children with type 2 and 3 spinal muscular atrophy

**DOI:** 10.1186/s11689-025-09592-x

**Published:** 2025-01-24

**Authors:** Huirong Nie, Shasha Lan, Huan Wang, Pei Xiang, Mengzhen Yan, Yang Fan, Wanqing Shen, Yijuan Li, Wen Tang, Zhiyun Yang, Yujian Liang, Yingqian Chen

**Affiliations:** 1 Department of Radiology, The First Affiliated Hospital of Sun Yat-sen University, No 58 Zhongshan 2nd Road, Guangzhou, 510080 China; 2 Department of Pediatric Intensive Care Unit, The First Affiliated Hospital of Sun Yat-sen University, No 58 Zhongshan 2nd Road, Guangzhou, 510080 China; 3 MR Research China, GE Healthcare, Beijing, China; 4 Department of Interventional Oncology, The First Affiliated Hospital of Sun Yat-sen University, Guangzhou, China

**Keywords:** Diffusion tensor imaging, Spinal muscular atrophy, Structural magnetic resonance imaging, White matter

## Abstract

**Background:**

Spinal muscular atrophy (SMA) is caused by reduced expression of survival motor neuron (SMN) protein. Previous studies indicated SMA causes not only lower motor neuron degeneration but also extensive brain involvement. This study aimed to investigate the changes of brain white matter and structural network using diffusion tensor imaging (DTI) in children with type 2 and 3 SMA.

**Methods:**

Forty-two type 2 and 3 pediatric SMA patients and 42 age- and gender-matched healthy controls (HC) were prospectively enrolled in this study. The tract-based spatial statistics (TBSS) was used to assess white matter integrity and the structural network properties were calculated based on DTI white matter fiber tracking and the graph theory approach. A partial correlation was performed to explore the relationship between white matter parameters and clinical characteristics.

**Results:**

In total, 42 patients (mean age, 10.86 ± 4.07 years; 23 men) were included. TBSS analysis revealed widespread white matter changes in SMA patients. The SMA patients showed changes in multiple small-world and network efficiency parameters. Compared to the HC group, SMA showed increased characteristic path length (L_p_), normalized clustering coefficient (γ), small-world characteristic (σ), and decreased global efficiency (E_glob_) (all *p* < 0.05). In the node properties, right supramarginal gyrus, right orbital part of superior frontal gyrus, right supplementary motor area, and left median cingulate and paracingulate gyri changed in SMA patients. A decreased axial diffusivity (AD) value was associated with lower Hammersmith Functional Motor Scale-Expanded scores (*r* = 0.45, *p* = 0.02), which means that the symptoms of SMA patients are more severe.

**Conclusions:**

This study found white matter and DTI-based brain network abnormalities in SMA patients, suggesting SMN protein deficiency may affect white matter development.

**Supplementary Information:**

The online version contains supplementary material available at 10.1186/s11689-025-09592-x.

## Introduction

Spinal muscular atrophy (SMA) is an autosomal recessive disease caused by mutations in survival motor neuron (SMN) 1 on chromosome 5, leading to reduced expression of SMN protein [1]. It is characterized by lower motor neuron degeneration [2]. Moreover, evidence in patients and animal models suggests that SMA is probably a multisystem disease affecting heart, kidney, liver and also brain in addition to the skeletal muscle [3].

The key contributing factor to the symptoms of SMA is the deficiency of survival motor neuron (SMN) protein [4]. Neuropathologic studies have shown that, besides the spinal cord, the SMN protein was particularly abundant in specific neuronal cell populations, such as in layer V pyramidal neurons of the neocortex, the pallidal neurons in the basal ganglia and the motor neurons of the brainstem [5]. These findings indicated that SMA could affect not only lower motor neuron but also the neuronal cells from the brain.

As advances in drugs, SMA patients are experiencing improved symptoms and longer life expectancy [6], which makes the study of the brain alterations of SMA patients increasingly important. It may potentially pave the way for new pathways of treatment or intervention for SMA patients. A few previous studies demonstrated morphological abnormalities of the brain for SMA patients [7–11]. Both cortical and sub-cortical changes were detected for different types of SMA patients. However, studies on changes in brain white matter affecting children’s motor and cognitive functions are still rarely reported. Moreover, current evidence in neuroimaging studies of SMA patients is limited to case reports with a high risk of bias in results [12]. Therefore, a large cohort should be investigated.

Previous studies have found structural brain abnormalities in patients with severe SMA like type 0 and 1 [7–9]. But the rapid deterioration progression of these two types may cause hypoxic/ischemic encephalopathy [13], which may mimic the structure alteration caused by the SMN deficit itself. The abnormal brain development in SMA patients with type 2 and 3 can reflect the real brain alteration caused by low-level SMN protein. So this study focused on brain white matter changes in patients with type 2 and 3 SMA.

DTI uses symmetric positive definite tensors from DWI to map the ellipsoidal water diffusion in brain tissue, reflecting white matter fiber structure. Several metrics derived from this tensor model, such as fractional anisotropy (FA), axial diffusivity (AD), radial diffusivity (RD), and mean diffusivity (MD), are commonly employed to represent the microstructure of brain anatomy. FA expresses the degree to which water diffusion is confined in one direction compared to others. The rise in FA with age in early life can be attributed to oligodendrocyte ensheathment surrounding axons. AD and RD represent the rate of microscopic water mobility parallel and perpendicular to the direction of axonal fibers in a regional tissue, respectively; they are commonly used to assess water content. MD represents the directionally averaged magnitude of water diffusion [14]. Graph theory simplifies the study of brain networks by modeling them as graphs with nodes for neural elements and edges for connections. The structural brain connectome based on the DTI approach reveals that human white matter networks exhibit a “small-world” architecture, characterized by dense local connections and sparse long-range connections, achieving a balance between local and global structural features [15].

Our study utilized diffusion MRI to investigate their alterations in brain white matter structure and network. Studying the brain white matter will help to characterize the new phenotype of SMA patients by comprehending the nature and extent of brain involvement in this disease and may expand our understanding of the disease’s pathophysiology. Moreover, the study of white matter networks will provide a holistic view to comprehend how SMA affects high-level activities like speech and cognition [16] and may be helpful for further treatment or interventions.

## Methods

### Participants

**Fig. 1:**
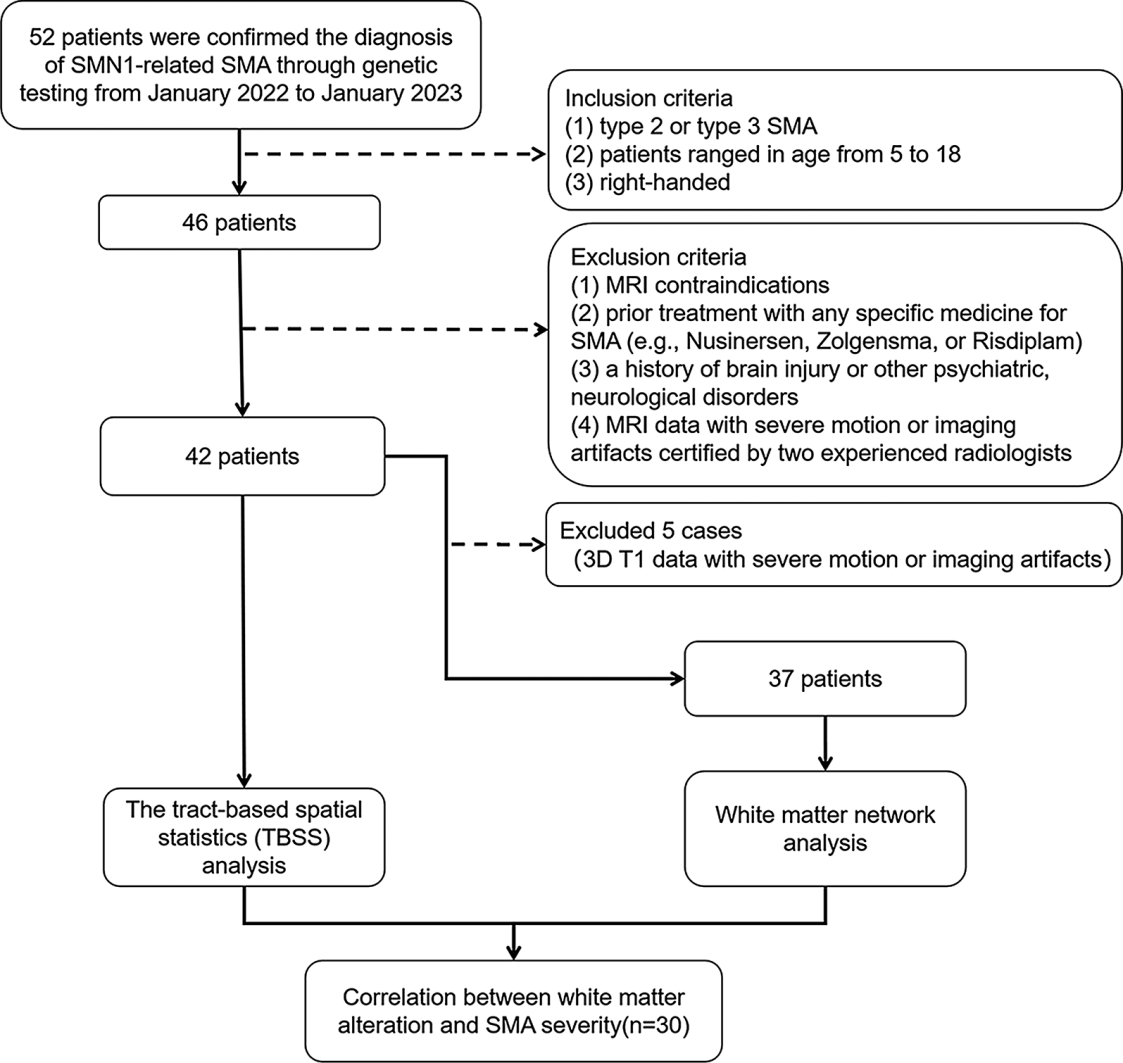
Flowchart of the whole workflow and recruitment of patients

This study was approved by Ethics Committee of the First Affiliated Hospital of Sun Yat-Sen University (No. [2021]710) and informed consent was obtained from the guardians of all subjects. All patients were prospectively recruited from the Department of Pediatric Intensive Care Unit (January 2022 to January 2023) and confirmed the diagnosis of SMN1-related SMA through genetic testing. The current consensus criteria were used for classification [17]. The following were the inclusion criteria: (1) type 2 or type 3 SMA [17]; (2) patients ranged in age from 5 to 18; (3) right-handed. Exclusion criteria included: (1) MRI contraindications; (2) prior treatment with any specific medicine for SMA (e.g., Nusinersen, Zolgensma, or Risdiplam); (3) a history of brain injury or other psychiatric, neurological disorders; and (4) MRI data with severe motion or imaging artifacts certified by two experienced radiologists. All HC were diagnosed by a clinical interview with an experienced pediatrician to rule out the possibility of developmental retardation, neurological disease, or any neuromuscular issue. A flowchart of the whole workflow is shown in Fig. 1.

### Clinical assessment

The standardized measure for assessing global motor impairment was the Hammersmith Functional Motor Scale-Expanded (HFMSE) scores [18]. The scores ranged from 0 to 66. A higher score reflected better motor function [19].

### Image acquisition

MRI examinations of all participants were performed on a 3.0T scanner (SIGNA Pioneer GE Healthcare, WI, USA) using a 32-channel head coil. Following localizers, a coronal T2-weighted sequence was scanned to rule out any cranial lesion. High-resolution three-dimensional(3D) T1-weighted anatomical images were acquired using the fast spoiled gradient recalled echo (FSPGR) sequence with the following parameters: repetition time (TR) = 7.5 ms, echo time (TE) = 3.1 ms, fip angle = 12°, 188 sagittal slices with slice thickness = 1 mm with no slice gap, a field of view (FOV) = 256 × 256 mm^2^, and data matrix = 256 × 256. DTI scans were obtained using a single-shot echo-planar imaging (EPI) sequence. Images slices were acquired parallel to the anterior-posterior commissure (AC-PC) to cover the entire brain with the following parameters: TR/TE = 10,000/83.9 ms; FOV = 224 × 224 mm^2^; matrix size = 112 × 112, resulting in a voxel size = 2 mm^3^; the number of excitations (NEX) = 1; number of diffusion encoded directions = 32; b-value = 1,000 s/mm^2^.

### Data processing

#### Data pre-processing

The DTI and 3D T1 images were pre-processed using the FSL (FMRIB Software Library, https://fsl.fmrib.ox.ac.uk/fsl/fslwiki/) and PANDA (https://www.nitrc.org/projects/panda/) softwares [20, 21]. Diffusion MRI data pre-processing steps were as follows: (1) all data were converted to NIfTI format, (2) head motion and eddy current correction, (3) gradient direction correction, (4) brain extraction, (5) diffusion tensor model fitting for the creation of fractional anisotropy (FA), mean diffusivity (MD), axial diffusivity (AD), and radial diffusivity (RD) parametric maps.

#### The tract-based spatial statistics (TBSS) analysis

For TBSS analysis, all participants’ FA images were non-linearly registered to the FA images of every other subject to obtain the most representative images. The FA images of each participant were registered to the most representative FA image through using FNIRT from FSL. And a white matter skeleton was generated from the FA maps. The aligned diffusion parametric maps of each participant, e.g. FA, MD, AD, RD maps, were projected onto the skeleton for between-group comparisons. Finally, the permutation tests by randomize order (5000 times) with threshold-free cluster enhancement (TFCE) based multiple comparison correction were conducted to test between-group differences.

#### Network construction

In the current study, whole-brain white matter tracts were reconstructed by deterministic fiber tractography, with angle > 45°or FA value < 0.2 as tracking termination condition. The automated anatomical labeling (AAL) atlas divided the whole brain into 90 cortical and subcortical regions, and nodes were defined as brain regions using the AAL atlas. Next, the number of white matter fiber tracts (FN) was calculated to define the network’s edges. Then, weighted and undirected symmetrical anatomical 90 × 90 matrices were obtained for each participant. After that, using 3 as the FN threshold to remove the false-positive connections in the network construction, the binarization brain structural network map was further constructed [22]. To rule out the spurious connections, connections that existed in less than 20% of the group subjects were excluded from further network properties analysis [23].

#### Network analysis

The GRETNA toolbox (http://www.nitrc.org/projects/gretna/) in MATLAB was used to calculate network properties [24]. Both global and nodal properties of network from each subject were obtained. Global metrics included small-world parameters, such as characteristic path length (L_p_), clustering coefficient (C_p_), normalized characteristic path length (λ), normalized clustering coefficient (γ), small-world characteristic (σ, σ = γ/λ), and network efficiency parameters, such as local efficiency (E_loc_), and global efficiency (E_glob_). The nodal properties included nodal degree, nodal efficiency, and nodal betweenness. The between-group differences of nodal profiles were presented using the BrainNet Viewer toolbox (https://www.nitrc.org/projects/bnv/).

### Statistical analysis

SPSS v25.0 (IBM Corp., Armonk, NY, USA) was used to compare clinical characteristics. The Shapiro–Wilk test was applied to verify whether the data met the conditions of the normal distribution. Two-sample t-test and Mann-Whitney U test were used to compare quantitative variables, and χ^2^ tests were used to compare qualitative variables. The results of TBSS were shown on the skeleton map after correction for multiple comparison with the TFCE technique. The Benjamini-Hochberg false-discovery rate (BHFDR) method was used to control for the error of multiple comparisons in the nodal properties of network. Partial correlation analyses were computed to examine relationships between HFMSE scores and brain white matter microstructure parameters, controlling for age and gender. A *p*-value < 0.05 was considered to be statistically significant.

## Results

### Demographic and clinical comparisons

In sum, 42 SMA patients (mean age ± standard deviation, 10.86 ± 4.07 years; 19 females) were enrolled in this study. For comparison, 42 age- and gender-matched healthy controls (HC) were included. Demographic and clinical statistics are shown in Table 1. A total of 30 patients’ HFMSE scores were collected. Due to the poor cooperation of some patients, the corresponding HFMSE score was not collected. There were no statistically significant differences between two groups in age and gender. (all *p* > 0.05).

**Table 1:** Demographic and clinical findings of SMA patients and healthy controls

Characteristics	SMA patients (*n* = 42)	Healthy controls (*n* = 42)	*p*-value
Age (mean ± SD)	10.86 ± 4.07	10.55 ± 2.57	0.85
Age range (years)	5–18	6–18	-
Gender (M/F) n% (M/F)	23/19 (54.76/45.24)	28/14 (66.67/33.33)	0.27
SMA type (2/3)	23/19	-	-
HFMSE scores Mean (SD) range	*n* = 30 28.23 (22.95) 0–65	-	-

HC, healthy controls; SMA, spinal muscular atrophy; SD, standard deviation; M, male; F, female; HFMSE, Hammersmith Functional Motor Scale-Expanded

### Abnormal white matter in TBSS results

**Fig. 2:**
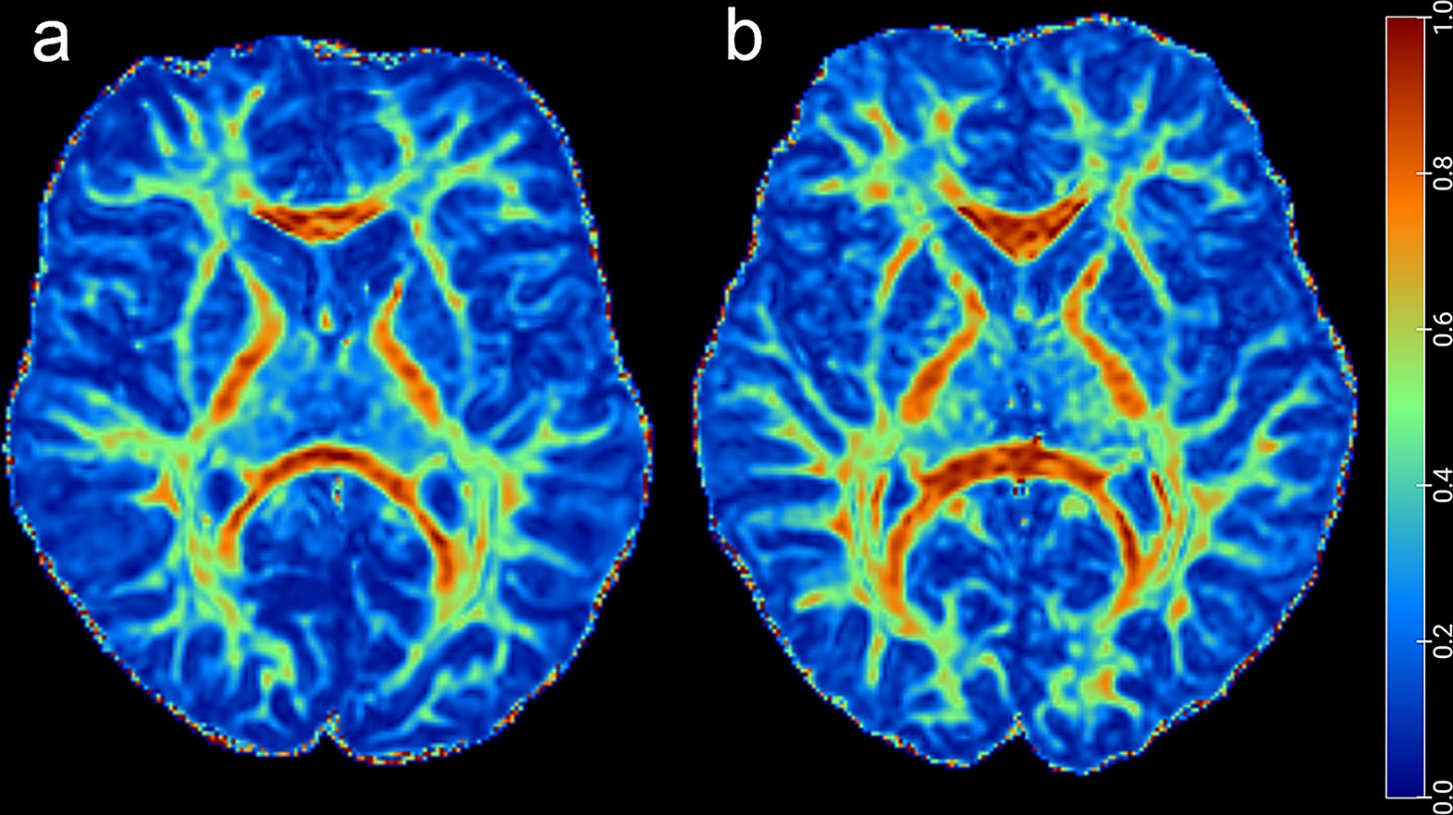
Comparison of brain FA images between an SMA patient and a healthy control child. **a** Brain FA image of an 11-year-old male SMA patient. **b** Brain FA image of a 10-year-old healthy control male child

**Fig. 3:**
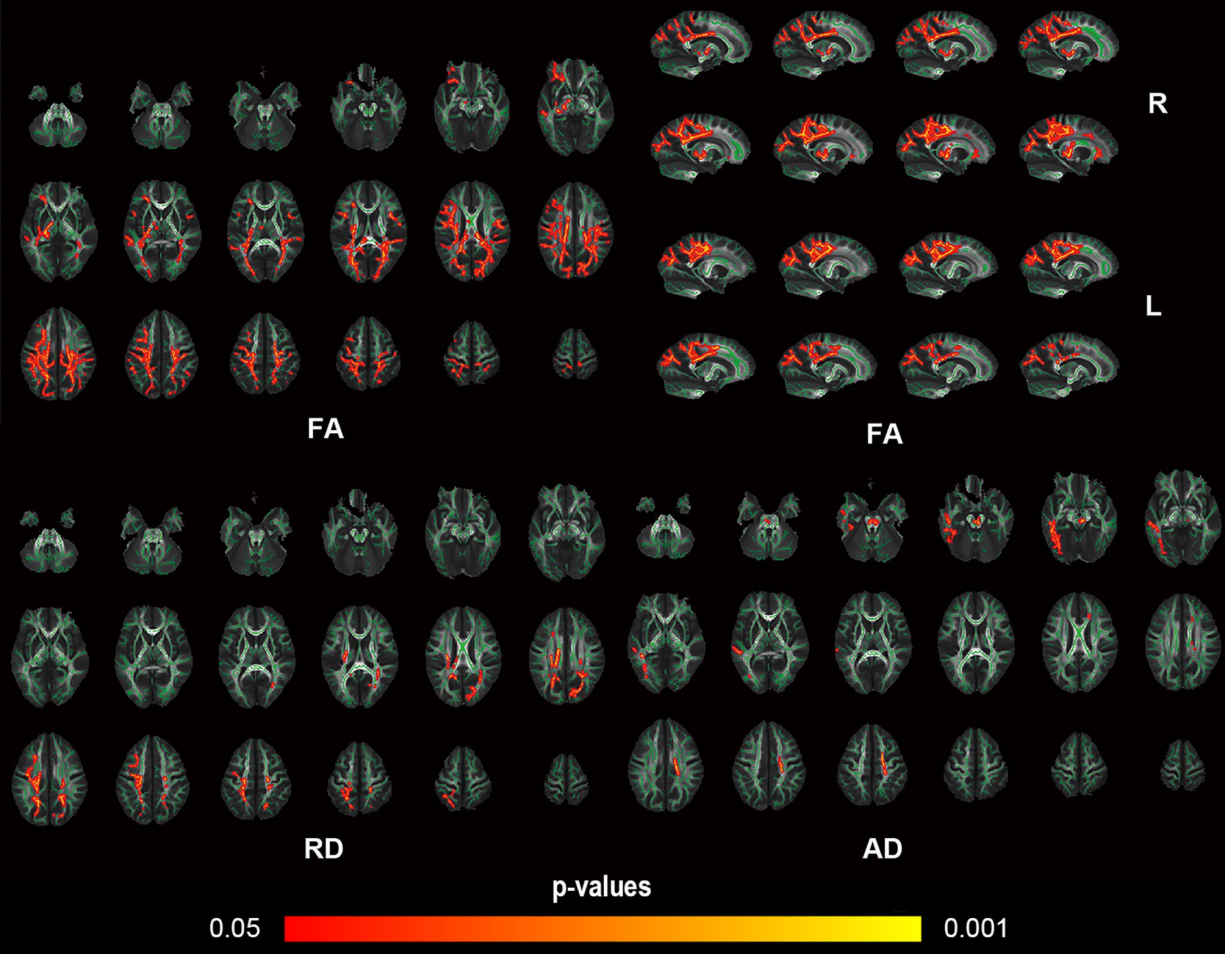
White matter brain regions with Between-group significant differences in FA, AD, RD values. White matter regions (red-yellow color) showed decreased FA and AD, and increased RD values in SMA group compared to the HC group (*p* < 0.05, TFCE and FWE correction). The white matter regions (green color) represented the white matter skeletons. FA, fractional anisotropy; AD, axial diffusivity; RD, radial diffusivity; SMA, spinal muscular atrophy; HC, healthy controls; L, left; R, right

We found widespread white matter alterations between SMA and HC groups among a lot of white matter fiber groups of the brain. Figure 2 shows a comparison of brain FA images between an SMA patient and a healthy control child, revealing decreased FA values in some white matter fibers of the SMA patient. Compared with the HC group, the FA value of projecting fibers, including the left part of corticospinal tract (CST) and bilateral anterior thalamic radiation, commissural fibers, such as the corpus callosum(CC), association fibers, including the bilateral superior and inferior longitudinal fasciculus(SLF and ILF), the bilateral Inferior fronto-occipital fasciculus(IFOF) and the left part of uncinate fasciculus and limbic system fibers, the bilateral cingulum, significantly decreased in the SMA group (*p* < 0.05, TFCE and FWE correction, Fig. 3). In comparison with HC group, the SMA group showed increased RD values within pathways that were mainly co-localized with the decreased FA values (*p* < 0.05, TFCE and FWE correction, Fig. 3). The AD value decreased in SMA group were localized in the bilateral CST, the bilateral anterior thalamic radiation, the left part of IFOF, the bilateral SLF and the left part of ILF (*p* < 0.05, TFCE and FWE correction, Fig. 3). There was no statistically significant difference in MD between the two groups.

### Global properties of the white matter networks

**Fig. 4:**
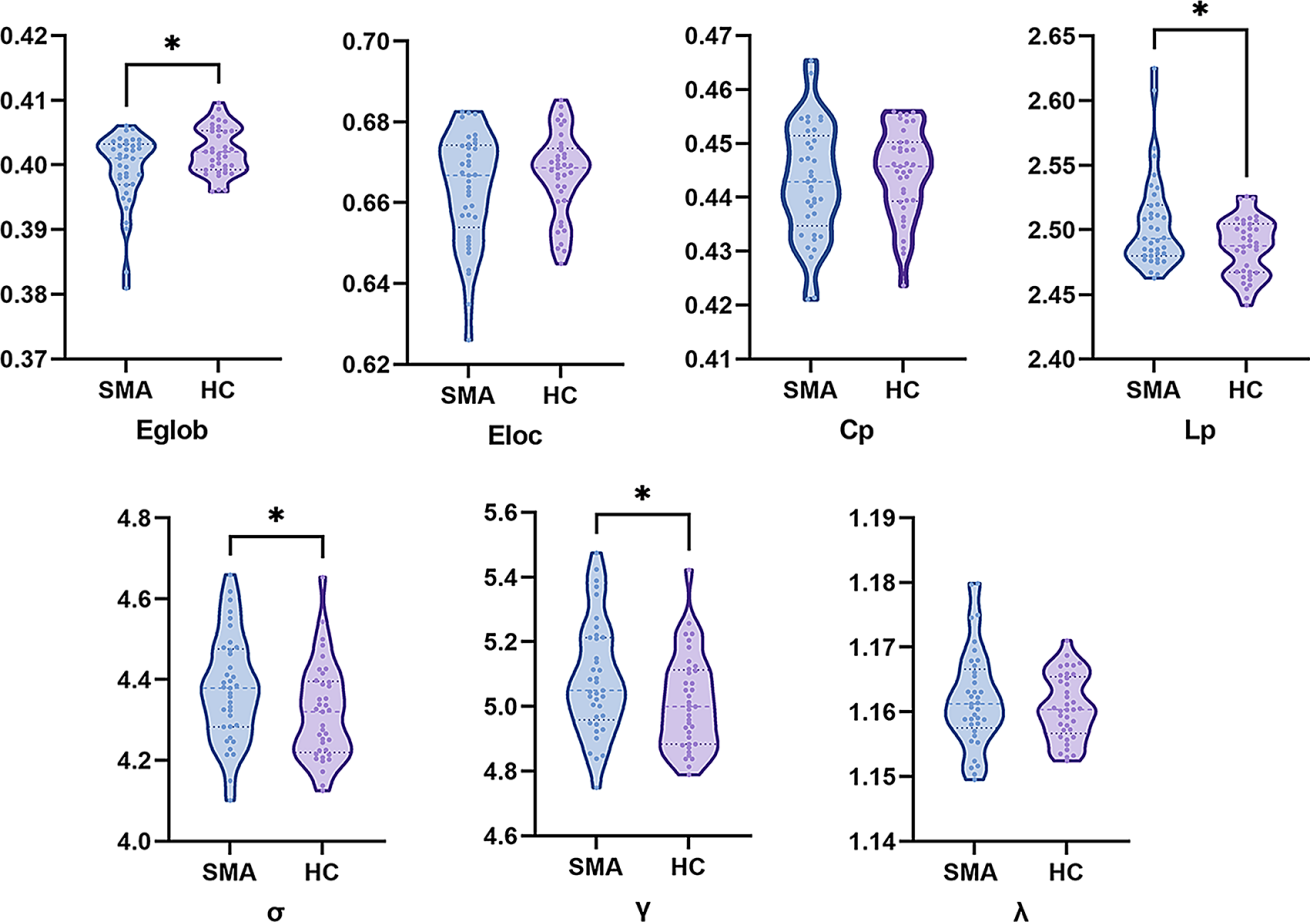
Between-group differences in the global parameters of brain white matter network. The SMA group showed a significant decrease in E_glob_ (*p* = 0.01) but significant increases in L_p_ (*p* = 0.01), γ (*p* = 0.0497), and σ (*p* = 0.047) compared with the HC group. E_glob_, global efficiency; E_loc_, local efficiency; C_p_, clustering coefficient; L_p_, characteristic path length; σ = γ/λ, small-world characteristic; λ, normalized characteristic path length; γ, normalized clustering coefficient; SMA, spinal muscular atrophy; HC, health controls; *p < 0.05

Subjects with artifacts in some 3D T1 images were excluded, and a total of 37 cases in the SMA group and 37 cases in the HC group were included in the analysis of white matter networks. Both the SMA group and HC group have the small world topology of the white matter brain networks (σ > 1). However, compared to the HC group, SMA showed increased L_p_ (*p* = 0.01), γ (*p* = 0.00497), and σ (*p* = 0.047), while there were no significant differences in the C_p_ (*p* = 0.45) and λ (*p* = 0.37). For network efficiency, the SMA group showed decreased E_glob_ (*p* = 0.01) compared with the HC group, with no significant differences in the E_loc_ (*p* = 0.23) (shown in Fig. 4).

### Nodal properties of the white matter networks

**Fig. 5:**
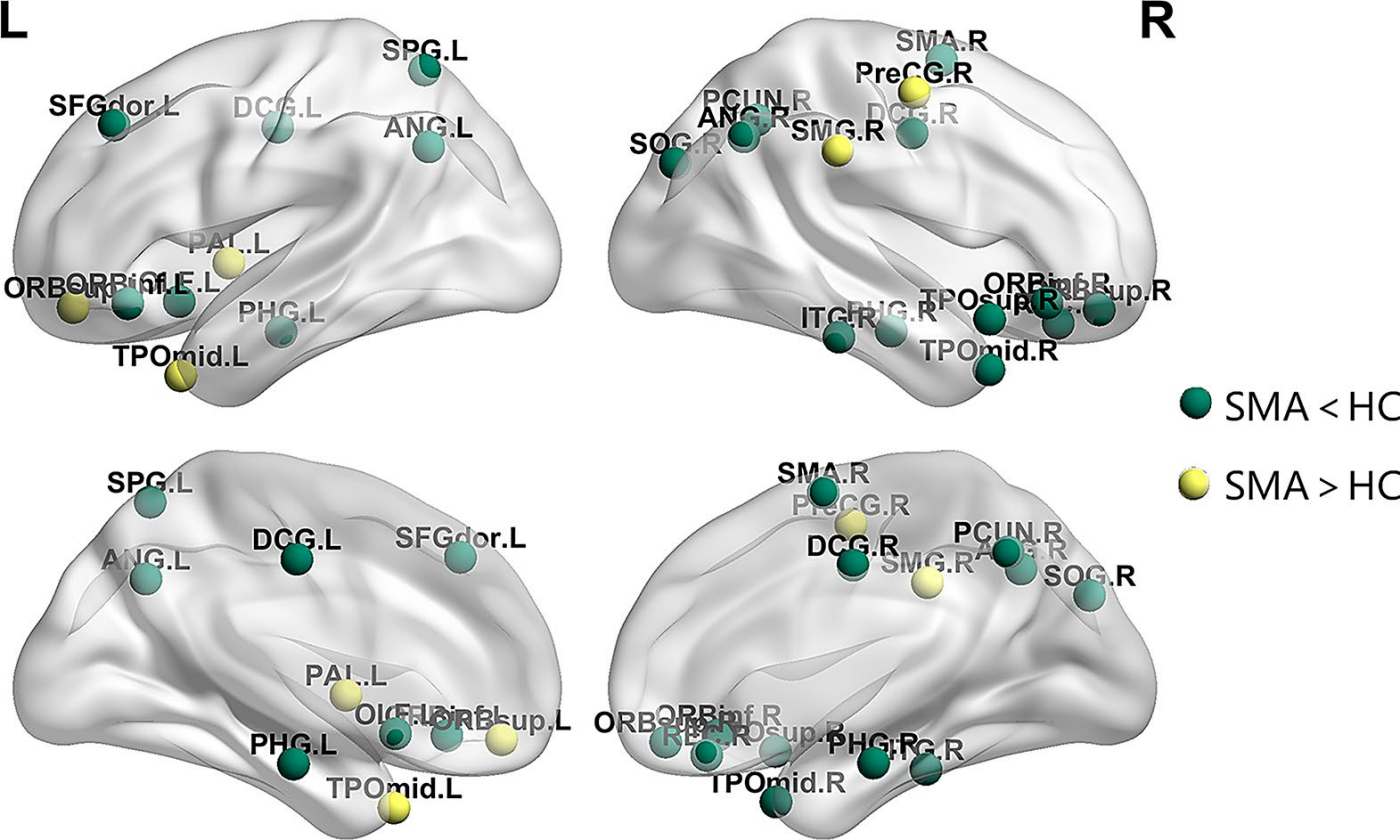
Regions of between-group differences in the nodal profiles of brain white matter network. It showed increased points (yellow) and decreased points (green) in the SMA group compared to the HC group. SMG, supramarginal gyrus; PreCG, precental gyrus; ORBinf, inferior frontal gyrus, orbital part; SOG, superior occipital gyrus; PAL, lenticular nucleus, pallidum; TPOmid, temporal pole: middle temporal gyrus; ORBsup, superior frontal gyrus, orbital part; SMA.R, right supplementary motor area; DCG, median cingulate and paracingulate gyri; SFGdor, superior frontal gyrus, dorsolateral; OLF, olfactory cortex; REC, gyrus rectus; PHG, parahippocampal gyrus; ANG, angular gyrus; ITG, inferior temporal gyrus; SPG, superior parietal gyrus; PCUN, precuneus; TPOsup, temporal pole: superior temporal gyrus; L, left; R, right; SMA, spinal muscular atrophy; HC, health controls

According to the AAL-90 atlas, compared to the HC group, the SMA group showed altered nodal profiles mainly in 5 categories: (1) the default mode network (DMN) that included the left dorsolateral of superior frontal gyrus (SFGdor.L), bilateral orbital part of inferior frontal gyrus (ORBinf), left olfactory cortex (OLF.L), right gyrus rectus (REC.R), bilateral parahippocampal gyrus (PHG), right supramarginal gyrus (SMG.R), bilateral angular gyrus (ANG), right precuneus (PCUN.R), right temporal pole: superior temporal gyrus (TPOsup.R), bilateral temporal pole: middle temporal gyrus (TPOmid); (2) the frontoparietal network (FPN) that included the bilateral orbital part of superior frontal gyrus (ORBsup), bilateral median cingulate and paracingulate gyri (DCG) and right inferior temporal gyrus (ITG.R); (3) the sensorimotor network (SEN) that included the right precental gyrus (PreCG.R), right supplementary motor area (SMA.R); (4) the visual network (VN) that included right superior occipital gyrus (SOG.R) and left superior parietal gyrus (SPG.L) and (5) the left pallidum of lenticular nucleus (PAL.L)(shown in Fig. 5). There were no significant differences in other nodal properties. However, after performing BenjaminiHochberg’s procedure, only right supramarginal gyrus (SMG.R), right orbital part of superior frontal gyrus (ORBsup.R), right supplementary motor area (SMA.R), and left median cingulate and paracingulate gyri (DCG.L) were statistically significant (*p* < 0.05, FDR corrected).

### Correlation between white matter alteration and SMA severity

**Fig. 6:**
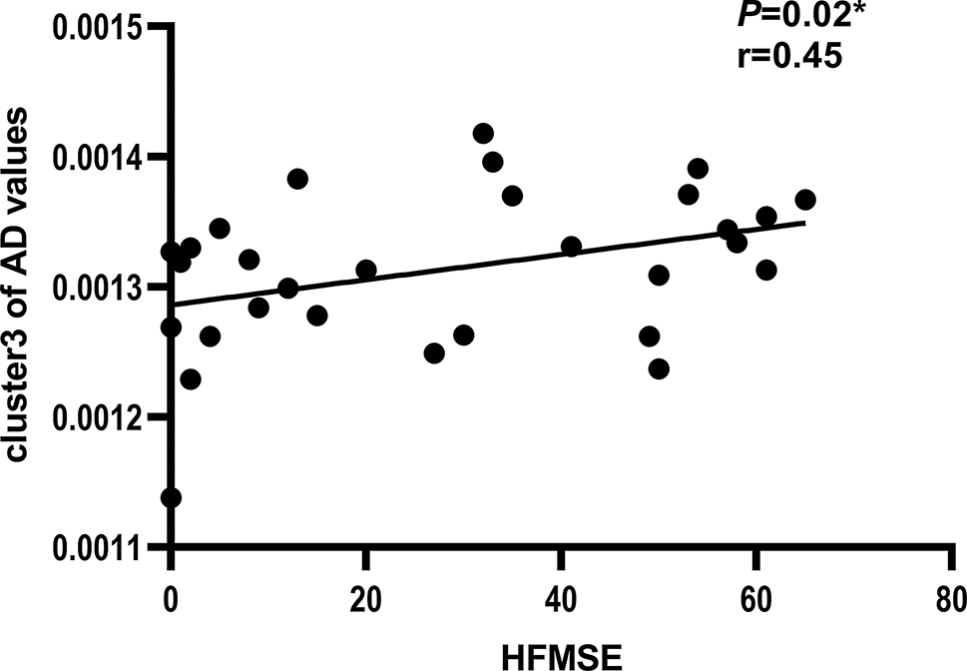
Relationship between the cluster 3 of AD values and the HFMSE in the SMA group. The results of correlation analysis between HFMSE scores (x-axis) and the cluster 3 of AD values (y-axis) (*r* = 0.45, *p* = 0.02). AD, axial diffusivity; HFMSE, Hammersmith Functional Motor Scale-Expanded; SMA, spinal muscular atrophy

The correlation analysis was used to explore the inner relationship between the above brain white matter microstructure parameters and symptom severity in SMA. Cluster 3 (included right anterior thalamic radiation, corticospinal tract and superior longitudinal fasciculus) of AD values was positively associated with HFMSE scores (*r* = 0.45, *p* = 0.02, shown in Fig. 6) in SMA. However, no significant correlations were found between other parameters and HFMSE scores.

## Discussion

This study demonstrated extensive brain white matter alterations in pediatric SMA types 2 and 3, which is responsive to the effects of SMN protein deficit on brain white matter since individuals with SMA types 2 and 3 are less likely to be affected by ischemic-hypoxic encephalopathy [13]. There are several important findings in this study. Firstly, we found widespread white-matter changes among many fiber groups of the brain, including projecting, commissural, association, and limbic system fibers. Secondly, there is a reduction in the ability to integrate and the efficiency of information transit across the entire brain network in SMA patients. Finally, at the nodal level, altered nodal profiles were mainly located in right supramarginal gyrus, right orbital part of superior frontal gyrus, right supplementary motor area, and left median cingulate and paracingulate gyri, which are known to be involved in cognitive function, emotion regulation, motor planning and execution, and verbal working memory [25–29].

### TBSS parameters abnormality

In SMA patients, widespread white matter alterations were shown in our study, with the parameters FA and AD of the SMA group being smaller than those of the HC group, and RD of the SMA group being larger than those of the HC group. A decrease in the FA value suggests a reduction in integrity and damage to the white matter structure, from gliosis, etc., axonal injury, and demyelination are among the potential causes. Also, diffusion along the axon direction decreases with a decreasing AD value, indicating axonal injury or the breakdown of fiber bundle consistency. Additionally, an increase in RD illustrates a loss of myelin or compromised integrity [14]. We propose that a key contributor to the cognitive impairments and impaired motor function linked to SMA is aberrant white matter fiber development. The corpus callosum has the function of transmitting information across the midline of the brain, which is associated with cognition, memory, and emotional modulation [30]. The left SLF-I is linked to verbal working memory, with SLF-III supporting the working memory phonological loop. Right hemisphere SLF-I/II damage impairs visuospatial working memory [31]. The functions of the corticospinal tract are the modulation of afferent signals, spinal reflexes, and motor neuron activation. The corticospinal tract holds a crucial position within the motor system, as it facilitates voluntary movements in the distal extremities [32]. We thought that abnormalities in the development of the corpus callosum, superior longitudinal fasciculus, and corticospinal tract might be associated with SMA-related cognitive function and motor function changes. A recent study showed that patients with type 2 and 3 SMA had significantly lower bilateral white matter volumes in the frontal and parietal lobes [33], which is consistent with our findings.

Besides, deterioration of clinical symptoms and muscle atrophy evaluated by HFMSE scores were found to be accompanied by decreased AD values in specific regions. The areas included right anterior thalamic radiation, corticospinal tract and superior longitudinal fasciculus, which are related to motor function [32] and executive function [31]. Changes in the brain white matter may be associated with decreased motor function due to the disease.

### Disrupted small-world networks

It is well-known that information transmission is the primary role of brain white matter. The aberrant development of white matter fibers usually causes abnormal white matter network integration, which is also seen in SMA patients. The SMA group had higher L_p_, γ, and σ and lower E_glob_ than the HC group. Lower E_glob_ and higher L_p_ indicate a decline in the ability for integration and the efficiency of information transport in parallel across the entire brain network [34]. It suggests that the SMA white matter network had a less optimal topological organization and a tendency to become high-cost and low-profit that more unlike a brain’s small world network [15, 35]. Therefore, SMA may cause dysfunctions of large-scale spatially distributed neural networks, particularly whole-brain integration deficits. This finding aids in the comprehension of SMA symptoms.

### Alterations in regional topology metrics

In a further analysis of node properties, multiple node properties were found to be different in the anatomical networks of the SMA group compared to the controls. Those brain regions were associated with alterations in cognitive function, emotion regulation, motor planning and execution, and verbal working memory [25–29]. For the cognitive function, several previous studies have identified varying degrees of cognitive dysfunction in patients with SMA [16, 36–39]. Disruption of the white matter microstructure of the brain has been reported to alter the cognitive network of the brain and is one of the key pathological underpinnings of cognitive impairment [40]. Further more in-depth studies following up SMA patients will be crucial to explain this finding. Previous study also has found that topological properties in the cortical-limbic-cerebellum circuit was disrupted in SMA [41]. In our study, the brain regions where node parameters have changed are widespread. We have not yet found a common neural pathway for the above different brain regions. They are involved in multiple neural networks, including the DMN, though their involvement varies. Overall, they support various cognitive, emotional, and motor functions. In the future, we hope to clarify the neural pathways that cause SMA patients’ diseases through further research.

Altered nodal metrics in SMA were found in right supplementary motor area, which is usually regarded as an essential region for motor planning and execution [25]. Abnormal alterations in this area suggested that brain involvement may be associated with symptoms of SMA, especially those of impaired motor function. In the node parameters, the abnormality of the supplementary motor area and the HFMSE scores are not correlated, which may not represent that the area is not correlated in the real situation, but only due to the limitation of the relatively indirect measurement method of network analysis. In the future, we will adopt more advanced methods for further research. With the advent of pharmacotherapy, motor function will be enhanced in SMA patients. Thus, further longitudinal studies are essential to determine whether connectivity in this region will improve after treatment.

### Reasons for abnormal parameters in white matter

The possible causes of abnormal white matter fibers in SMA patients are complex. Firstly, low-level SMN protein caused by genetic mutations is believed to have a significant impact on the brain. Generally, all major white matter tracts are present by the end of normal gestation (37–42 weeks). Processes including myelination and synaptogenesis occur rapidly during the first 2–3 years of life, and ongoing brain remodeling continues into early adulthood [42]. The observed alteration in white matter is probably the result of a delated myelin maturation throughout development.

Also, the impact of limb disuse on the brain cannot be ignored. In the other functional neurological disorders, subtle changes in brain area volume and cortical thickness were also found and were considered to be secondary changes due to limb disuse in the case of thalamic or motor areas [43]. Similarly, the limb disuse due to muscle weakness in SMA patients should also be considered as an influencing factor of white matter changes.

Besides, the age-related changes may have a small impact on the results. But it has been minimized to a relatively low level by matching the age and gender of the participants and enrolling participants aged 5 to 18 who were in a relatively stable stage of brain development.

### Study limitation

This study also has a few limitations. First, this was a single-center study with a small number of participants and a limited sample size due to the low morbidity of SMA. Second, the study used a cross-sectional design. A longitudinal design is a natural extension of our proof-of-concept findings. Third, due to failure to collect scales for patient cognitive assessment, we did not capture correlations between neuroimaging measures and cognitive assessments. In the future, multi-center research with large sample sizes and even longitudinal studies combining structural and functional MRI may allow for a deeper comprehension of the neurobiological aspects of SMA.

## Conclusion

In conclusion, this study discovered extensive brain white matter and DTI-based brain network alterations in pediatric SMA type 2 and 3, which indicate that SMN protein deficit may cause abnormal development of white matter in the brain of SMA. So, more attention should be paid to the brain involvement of SMA patients to help them reintegrate into society.

## Electronic supplementary material

Below is the link to the electronic supplementary material.

Supplementary Material 1

## Data Availability

No datasets were generated or analysed during the current study.

## Abbreviations

SMA: Spinal muscular atrophy
DTI: Diffusion tensor imaging
FA: Fractional anisotropy
MD: Mean diffusivity
AD: Axial diffusivity
RD: Radial diffusivity
L_p_: Characteristic path length
C_p_: Clustering coefficient
λ: Normalized characteristic path length
γ: Normalized clustering coefficient
σ: Small-world characteristic
E_loc_: Local efficiency
E_glob_: Global efficiency
HFMSE: Hammersmith Functional Motor Scale-Expanded
