# Supplementary material for: Reduced white matter integrity and disrupted brain network in children with type 2 and 3 spinal muscular atrophy
Source: J Neurodev Disord. 2025 Jan 24;17:3. doi: 10.1186/s11689-025-09592-x (PMC11761759; doi:10.1186/s11689-025-09592-x)
Supplement: Supplementary file 1 — Supplementary Material 1 [file 11689_2025_9592_MOESM1_ESM.docx]

**Supplementary materials**

Table 1. White matter brain regions with significant differences in FA values between the SMA group and the HC group

| Cluster | Cluster size | MNI center of mass | | | *p*-value | | Brain regions | Side | |
| --- | --- | --- | --- | --- | --- | --- | --- | --- | --- |
|  |  | X | Y | Z |  |  |  |  |  |
| 1 | 13633 | -25 | -31 | 35 | 0.001 | Anterior thalamic radiation | | | L |
|  |  |  |  |  |  | Corticospinal tract | | | L |
|  |  |  |  |  |  | Cingulum (cingulate gyrus) | | | L |
|  |  |  |  |  |  | Cingulum (hippocampus) | | | L |
|  |  |  |  |  |  | Forceps major | | | - |
|  |  |  |  |  |  | Forceps minor | | | - |
|  |  |  |  |  |  | Inferior fronto-occipital fasciculus | | | L |
|  |  |  |  |  |  | Inferior longitudinal fasciculus | | | L |
|  |  |  |  |  |  | Superior longitudinal fasciculus | | | L |
|  |  |  |  |  |  | Uncinate fasciculus | | | L |
|  |  |  |  |  |  | Superior longitudinal fasciculus (temporal part) | | | L |
| 2 | 9190 | 27 | -52 | 30 | 0.003 | Anterior thalamic radiation | | | R |
|  |  |  |  |  |  | Corticospinal tract | | | R |
|  |  |  |  |  |  | Cingulum (cingulate gyrus) | | | L |
|  |  |  |  |  |  | Cingulum (cingulate gyrus) | | | R |
|  |  |  |  |  |  | Cingulum (hippocampus) | | | R |
|  |  |  |  |  |  | Forceps major | | | - |
|  |  |  |  |  |  | Inferior fronto-occipital fasciculus | | | R |
|  |  |  |  |  |  | Inferior longitudinal fasciculus | | | R |
|  |  |  |  |  |  | Superior longitudinal fasciculus | | | R |
|  |  |  |  |  |  | Superior longitudinal fasciculus (temporal part) | | | R |
| 3 | 526 | -54 | -33 | 1 | 0.037 | Inferior longitudinal fasciculus | | | L |
|  |  |  |  |  |  | Superior longitudinal fasciculus | | | L |
|  |  |  |  |  |  | Superior longitudinal fasciculus (temporal part) | | | L |
| 4 | 101 | -34 | -60 | 29 | 0.048 | Inferior longitudinal fasciculus | | | L |
|  |  |  |  |  |  | Superior longitudinal fasciculus | | | L |

FA, fractional anisotropy; HC, healthy controls; SMA, spinal muscular atrophy; L, left; R, right.

Table 2. White matter brain regions with significant differences in AD values between the SMA group and the HC group

| Cluster | Cluster size | | MNI center of mass | | | *p*-value | Brain regions | Side |
| --- | --- | --- | --- | --- | --- | --- | --- | --- |
|  |  |  | X | Y | Z |  |  |  |
| 1 | | 1156 | -50 | -39 | -17 | 0.036 | Inferior fronto-occipital fasciculus | L |
|  | |  |  |  |  |  | Inferior longitudinal fasciculus | L |
|  | |  |  |  |  |  | Superior longitudinal fasciculus | L |
|  | |  |  |  |  |  | Superior longitudinal fasciculus (temporal part) | L |
| 2 | | 535 | -54 | -33 | 1 | 0.041 | Inferior fronto-occipital fasciculus | L |
|  | |  |  |  |  |  | Inferior longitudinal fasciculus | L |
|  | |  |  |  |  |  | Superior longitudinal fasciculus | L |
|  | |  |  |  |  |  | Uncinate fasciculus | L |
|  | |  |  |  |  |  | Superior longitudinal fasciculus (temporal part) | L |
| 3 | | 495 | 19 | -8 | 45 | 0.038 | Anterior thalamic radiation | R |
|  | |  |  |  |  |  | Corticospinal tract | R |
|  | |  |  |  |  |  | Superior longitudinal fasciculus | R |
| 4 | | 308 | 1 | -25 | -14 | 0.041 | Anterior thalamic radiation | L |
|  | |  |  |  |  |  | Anterior thalamic radiation | R |
|  | |  |  |  |  |  | Corticospinal tract | L |
|  | |  |  |  |  |  | Corticospinal tract | R |

AD, axial diffusivity; HC, healthy controls; SMA, spinal muscular atrophy; L, left; R, right.

Table 3. White matter brain regions with significant differences in RD values between the SMA group and the HC group

| Cluster | Cluster size | | MIN center of mass | | | *p*-value | Brain regions | Side |
| --- | --- | --- | --- | --- | --- | --- | --- | --- |
|  |  |  | X | Y | Z |  |  |  |
| 1 | | 3368 | -20 | -31 | 34 | 0.018 | Anterior thalamic radiation | L |
|  | |  |  |  |  |  | Corticospinal tract | L |
|  | |  |  |  |  |  | Cingulum (cingulate gyrus) | L |
|  | |  |  |  |  |  | Cingulum (hippocampus) | L |
|  | |  |  |  |  |  | Forceps major | - |
|  | |  |  |  |  |  | Inferior fronto-occipital fasciculus | L |
|  | |  |  |  |  |  | Inferior longitudinal fasciculus | L |
|  | |  |  |  |  |  | Superior longitudinal fasciculus | L |
|  | |  |  |  |  |  | Superior longitudinal fasciculus (temporal part) | L |
| 2 | | 756 | 22 | -49 | 34 | 0.033 | Anterior thalamic radiation | R |
|  | |  |  |  |  |  | Cingulum (cingulate gyrus) | R |
|  | |  |  |  |  |  | Cingulum (hippocampus) | R |
|  | |  |  |  |  |  | Forceps major | - |
|  | |  |  |  |  |  | Inferior fronto-occipital fasciculus | R |
|  | |  |  |  |  |  | Inferior longitudinal fasciculus | R |
|  | |  |  |  |  |  | Superior longitudinal fasciculus | R |
|  | |  |  |  |  |  | Superior longitudinal fasciculus (temporal part) | R |
| 3 | | 538 | 18 | -75 | 25 | 0.04 | Anterior thalamic radiation | R |
|  | |  |  |  |  |  | Forceps major | - |
|  | |  |  |  |  |  | Inferior fronto-occipital fasciculus | R |
|  | |  |  |  |  |  | Inferior longitudinal fasciculus | R |
| 4 | | 120 | 24 | -29 | 44 | 0.046 | Corticospinal tract | R |
|  | |  |  |  |  |  | Superior longitudinal fasciculus | R |

RD, radial diffusivity; HC, healthy controls; SMA, spinal muscular atrophy; L, left; R, right.

Table 4. Comparison of global network metrics between the SMA group and the HC group

|  | SMA | HC | t-value | *p~~P~~*-value |
| --- | --- | --- | --- | --- |
| Small world |  |  |  |  |
| σ | 4.378±0.132 | 4.319±0.118 | 2.025 | 0.047* |
| λ | 1.162±0.008 | 1.161±0.005 | 0.902 | 0.370 |
| γ | 5.088±0.175 | 5.013±0.146 | 1.996 | < 0.050*^†^ |
| L_p_ | 2.505±0.037 | 2.487±0.022 | 2.624 | 0.011* |
| C_p_ | 0.443±0.011 | 0.445±0.008 | -0.767 | 0.446 |
| Global efficiency |  |  |  |  |
| E_glob_ | 0.399±0.006 | 0.402±0.004 | -2.629 | 0.010* |
| E_loc_ | 0.663±0.014 | 0.667±0.011 | -1.225 | 0.225 |

Mean ± SD, SD standard deviation; SMA, spinal muscular atrophy; HC, healthy controls; E_glob_, global efficiency; E_loc_, local efficiency; C_p_, clustering coefficient; L_p_, characteristic path length; σ = γ/λ, small-world characteristic; λ, normalized characteristic path length; γ, normalized clustering coefficient; SMA, spinal muscular atrophy; HC, health controls. **p~~P~~* < 0.05. †*p~~P~~* = 0.0497.

Table 5. Regions showing altered nodal metrics in the SMA group and the HC group

| Brain regions | Nodal degree | | Nodal efficiency | | Nodal betweenness | |
| --- | --- | --- | --- | --- | --- | --- |
|  | *p~~P~~*-value | *p~~P~~*-value (FDR-corrected) | *p~~P~~*-value | *p~~P~~*-value (FDR-corrected) | *p~~P~~*-value | *p~~P~~*-value (FDR-corrected) |
| SMA＞HC |  |  |  |  |  |  |
| PreCG.R | 0.010* | 0.142 | 0.168 | 0.410 | 0.002* | 0.074 |
| ORBinf.L | 0.494 | 0.886 | 0.406 | 0.589 | 0.043* | 0.651 |
| SMG.R | 0.003* | 0.056 | 0.012* | 0.230 | 0.000* | 0.010** |
| PAL.L | 0.812 | 0.988 | 0.987 | 0.988 | 0.049* | 0.651 |
| TPOmid.L | 0.580 | 0.954 | 0.506 | 0.699 | 0.040* | 0.651 |
| SMA＜HC |  |  |  |  |  |  |
| SFGdor.L | 0.030* | 0.197 | 0.124 | 0.386 | 0.341 | 0.730 |
| ORBsup.L | 0.020* | 0.197 | 0.005* | 0.216 | 0.069 | 0.651 |
| ORBsup.R | 0.001* | 0.030** | 0.018* | 0.230 | 0.157 | 0.651 |
| ORBinf.R | 0.027* | 0.197 | 0.073 | 0.342 | 0.440 | 0.861 |
| SMA.R | 0.001* | 0.030** | 0.002* | 0.210 | 0.908 | 0.982 |
| OLF.L | 0.029* | 0.197 | 0.113 | 0.386 | 0.735 | 0.955 |
| REC.R | 0.042* | 0.255 | 0.117 | 0.386 | 0.327 | 0.719 |
| DCG.L | 0.001* | 0.030** | 0.025* | 0.244 | 0.198 | 0.651 |
| DCG.R | 0.018* | 0.197 | 0.142 | 0.386 | 0.284 | 0.672 |
| PHG.L | 0.005* | 0.087 | 0.017* | 0.230 | 0.163 | 0.651 |
| PHG.R | 0.130 | 0.559 | 0.030* | 0.244 | 0.267 | 0.651 |
| SOG.R | 0.149 | 0.580 | 0.013* | 0.230 | 0.033* | 0.651 |
| SPG.L | 0.070 | 0.350 | 0.039* | 0.250 | 0.060 | 0.651 |
| ANG.L | 0.471 | 0.865 | 0.048* | 0.285 | 0.414 | 0.827 |
| ANG.R | 0.029* | 0.197 | 0.022* | 0.243 | 0.236 | 0.651 |
| PCUN.R | 0.083 | 0.392 | 0.037* | 0.250 | 0.265 | 0.651 |
| TPOsup.R | 0.057 | 0.300 | 0.038* | 0.250 | 0.880 | 0.970 |
| TPOmid.R | 0.011* | 0.142 | 0.029* | 0.244 | 0.538 | 0.876 |
| ITG.R | 0.031* | 0.197 | 0.013* | 0.230 | 0.565 | 0.891 |

The brain regions were defined by AAL. SMA, spinal muscular atrophy; HC, healthy controls; L, left; R, right; *Uncorrected *p~~P~~* < 0.05; ***p~~P~~* < 0.05 after performing the Benjamini-Hochberg false discovery rate correction; SMG, supramarginal gyrus; PreCG, precental gyrus; ORBinf, inferior frontal gyrus, orbital part; SOG, superior occipital gyrus; PAL, lenticular nucleus, pallidum; TPOmid, temporal pole: middle temporal gyrus; ORBsup, superior frontal gyrus, orbital part; SMA.R, right supplementary motor area; DCG, median cingulate and paracingulate gyri; SFGdor, superior frontal gyrus, dorsolateral; ORBinf, inferior frontal gyrus, orbital part; OLF, olfactory cortex; REC, gyrus rectus; PHG, parahippocampal gyrus; ANG, angular gyrus; ITG, inferior temporal gyrus; SPG, superior parietal gyrus; PCUN precuneus; TPOsup, temporal pole: superior temporal gyrus.
